# Deep Learning and fMRI-Based Pipeline for Optimization of Deep Brain Stimulation During Parkinson’s Disease Treatment: Toward Rapid Semi-Automated Stimulation Optimization

**DOI:** 10.1109/JTEHM.2024.3448392

**Published:** 2024-08-22

**Authors:** Jianwei Qiu, Afis Ajala, John Karigiannis, Jürgen Germann, Brendan Santyr, Aaron Loh, Luca Marinelli, Thomas Foo, Radhika Madhavan, Desmond Yeo, Alexandre Boutet, Andres Lozano

**Affiliations:** GE HealthCare Technology & Innovation Center Niskayuna NY 12309 USA; GE Global Research Niskayuna NY 12309 USA; Division of NeurosurgeryDepartment of SurgeryUniversity Health Network 7989 Toronto ON M5G 2C4 Canada; Division of NeurosurgeryDepartment of SurgeryUniversity of Toronto 7938 Toronto ON M5G 2C4 Canada; Joint Department of Medical ImagingUniversity of Toronto 7938 Toronto M5S 1A1 Canada; Krembil Brain Institute Toronto ON M5T 1M8 Canada; Center for Advancing Neurotechnological Innovation to Application (CRANIA) Toronto ON M5S 1A4 Canada

**Keywords:** Deep brain stimulation optimization, deep learning, fMRI, Parkinson’s disease, unsupervised feature extraction.

## Abstract

Objective: Optimized deep brain stimulation (DBS) is fast becoming a therapy of choice for the treatment of Parkinson’s disease (PD). However, the post-operative optimization (aimed at maximizing patient clinical benefits and minimizing adverse effects) of all possible DBS parameter settings using the standard-of-care clinical protocol requires numerous clinical visits, which substantially increases the time to optimization per patient (TPP), patient cost burden and limit the number of patients who can undergo DBS treatment. The TPP is further elongated in electrodes with stimulation directionality or in diseases with latency in clinical feedback. In this work, we proposed a deep learning and fMRI-based pipeline for DBS optimization that can potentially reduce the TPP from ~1 year to a few hours during a single clinical visit.Methods and procedures: We developed an unsupervised autoencoder (AE)-based model to extract meaningful features from 122 previously acquired blood oxygenated level dependent (BOLD) fMRI datasets from 39 a priori clinically optimized PD patients undergoing DBS therapy. The extracted features are then fed into multilayer perceptron (MLP)-based parameter classification and prediction models for rapid DBS parameter optimization.Results: The AE-extracted features of optimal and non-optimal DBS were disentangled. The AE-MLP classification model yielded accuracy, precision, recall, F1 score, and combined AUC of 0.96 ± 0.04, 0.95 ± 0.07, 0.92 ± 0.07, 0.93 ± 0.06, and 0.98 respectively. Accuracies of 0.79 ± 0.04, 0.85 ± 0.04, 0.82 ± 0.05, 0.83 ± 0.05, and 0.70 ± 0.07 were obtained in the prediction of voltage, frequency, and x-y-z contact locations, respectively.Conclusion: The proposed AE-MLP models yielded promising results for fMRI-based DBS parameter classification and prediction, potentially facilitating rapid semi-automated DBS parameter optimization. Clinical and Translational Impact Statement—A deep learning-based pipeline for semi-automated DBS parameter optimization is presented, with the potential to significantly decrease the optimization duration per patient and patients' financial burden while increasing patient throughput.

## I. Introduction

Deep brain stimulation (DBS) is a neurosurgical procedure that involves delivering a constant electric pulse using surgically implanted electrodes in specific target areas of the brain to suppress aberrant neural activities and/or modulate brain networks [1]. DBS procedures are routinely adopted for the treatment of movement disorders such as Parkinson’s disease (PD), essential tremor, and dystonia [2], [3], [4], and they have additionally shown promising results for a range of psychiatric, cognitive, pain, and seizure disorders [2], [5], [6]. The successful treatment of PD using sub-thalamic nucleus (STN) or globus pallidus internus (GPi) DBS hinges on precise surgical implantation and determining a patient-specific optimal combination of DBS parameters, including signal frequency, voltage, pulse width, and electrode contact location. DBS parameters are said to be optimized if they achieve maximal patient benefit while minimizing adverse effects [7]. It is well known that suboptimal DBS programming can lessen treatment efficacy, increase patient side effects, and drain the implanted pulse generator battery more quickly than necessary [8].

Based on the current standard-of-care empirical DBS programming, the search for an optimal combination of DBS parameters usually involves multiple time-consuming programming sessions. These multiple sessions substantially increase the time to optimization per patient (TPP) – averaging about 1 year – and impose a significant cost burden on patients. Additionally, the longer TPP leads to patient fatigue and ultimately limit the number of patients who can undergo DBS treatment [9], [10], [11]. Furthermore, the advent of newer DBS electrodes with an even greater number of directional contact locations makes the standard-of-care optimization method increasingly difficult as the expanded DBS parameter space has rendered it intractable for clinicians to empirically program the electrode within a clinically acceptable timeframe. This increased complication and difficulty has hindered the adoption of the newer and more effective DBS electrodes by clinicians [12], [13], [14], [15], [16]. In diseases such as dystonia, addiction, and depression, clinically based programming is further complicated as there is a latency – potentially in the order of weeks – between stimulation adjustments and subsequent clinical effect [17]. Due to such difficulties, an estimated 230,000 patients worldwide have undergone DBS therapy for a variety of neurological and non-neurological conditions. This number, although significant, is small compared to the eligible patient population for DBS [18].

The possibility of a biomarker-based DBS parameter optimization, which can substantially reduce the TPP during DBS therapy, has been previously established by our research team [11], [17]. Optimal DBS parameter settings were shown to yield unique functional magnetic resonance imaging (fMRI) response maps in PD patients undergoing DBS therapy. Blood oxygenation level dependent (BOLD) DBS-fMRI response maps associated with optimal stimulation of the left STN showed significant deactivations in the ipsilateral motor cortex, contra-lateral cerebellum, and orbito-lateral cortex, as well as significant activation in the ipsilateral thalamus. Subtherapeutic voltages triggered a decrease in the magnitude of the BOLD changes with a preserved topographic pattern. Supratherapeutic voltages yielded a relatively stronger BOLD response in the ipsilateral motor cortex and contra-lateral cerebellum and was also accompanied by increased BOLD signal in non-motor regions, such as the inferior frontal and occipital lobes.

Due to the uniqueness of the DBS-fMRI response pattern at optimal DBS parameter settings, features that characterize the patient’s optimal DBS parameters can be extracted from the fMRI response map and used to train deep learning (DL)-based DBS parameter classification and prediction models. Such DL models can facilitate semi-automated and rapid DBS parameter optimization that is unlike the current time-consuming standard-of-care optimization protocol. The feature extraction from the DBS-fMRI response maps can be carried out using pre-defined parcels in brain regions of motor function relevance as previously published in the parcel-based linear discriminant analyses (PB-LDA) method [11]. However, a more resilient feature extraction from the DBS-fMRI response maps in an unsupervised manner is needed to maximize the efficacy of a rapid DL-based DBS parameter optimization method. If robust enough, such unsupervised feature extraction model will also ensure that disentangled features of optimal and non-optimal stimulation are extracted from DBS-fMRI response maps that may contain minimal changes in the response pattern – due to differences in disease condition or changes in the stimulation side – without the need to retrain the feature extraction model.

In this work, we build and test a DL-based pipeline for optimization of DBS parameter settings. The pipeline uses features extracted from fMRI response maps of PD patients undergoing DBS therapy for model training and testing. We develop an unsupervised autoencoder (AE)-based feature extraction method to obtain latent features from the DBS-fMRI response maps. The learned features are subsequently used to train multilayer perceptron (MLP)-based DBS parameters classification and prediction models. Furthermore, we investigate the ability of the AE-based feature extraction model to extract disentangled features of optimal and non-optimal stimulation from DBS-fMRI response maps that contain changes in the response pattern that may be due to differences in stimulation side and/or PD disease condition.

## II. Methods and Procedures

### A. Experimental Data Description

**FIGURE 1.**
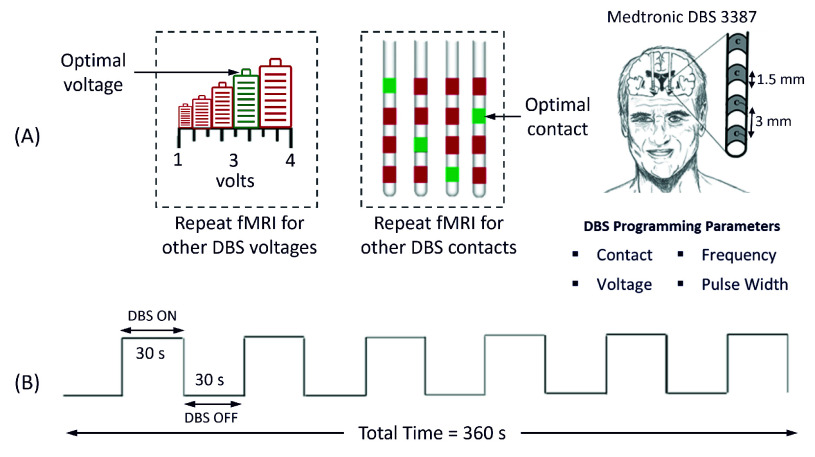
(A) The acquired data consisted of fMRI data from left stimulated PD patients at optimal and non-optimal contacts or voltages. (B) FMRI data were acquired using a 30 seconds DBS ON/OFF cycling paradigm for 360 seconds. The sketch of the human head was adapted from Boutet et al., 2021 [11], as permitted under the Creative Commons Attribution 4.0 International License.

In this work, we used our previous BOLD fMRI data acquired on a 3T GE HDx MRI scanner (GE HealthCare, Wisconsin, USA) from 39 PD patients (n =35 STN-DBS, n =4 GPi-DBS, mean age $= 62.4~\pm ~7.1$, 20 males, 19 females; study #NCT03153670) who had undergone left DBS treatment at Toronto Western Hospital. The optimal DBS parameters of all 39 patients was previously determined via the standard-of-care clinical optimization protocol [3], [4], and fMRI data was then acquired from all 39 PD patients with different DBS settings amounting to a total of 122 response maps. Data were acquired after protocols were approved by the institutional research ethics board at the University Health Network, #14-8255. All participants provided written informed consent prior to MRI scans, and a member of the clinical team was present to monitor patients during the MRI sessions. Patients were instructed to take their last dose of PD medication at least 24 hours before the study to avoid confounding responses. Other details of the patient demographics and DBS-fMRI data used in this work can be found in a previous publication [11]. The BOLD fMRI design implemented in the study data was aimed at distinguishing patterns of brain activation at optimal and non-optimal DBS parameter settings. DBS-fMRI experimental data included 6.5-minute fMRI sessions using a 30 second DBS-ON/OFF cycling paradigm as shown in Figure 1. All DBS-fMRI response maps correspond to different DBS parameter sets and were labeled as optimal or non-optimal by a movement disorder clinician based on optimal DBS parameter settings obtained via the standard-of-care clinical optimization protocol [3], [4]. The acquired axial 3D anatomical T1-weighted images were also used for rigid-body registration. Other details of the DBS-fMRI and anatomical data acquisition parameters have been previously published [11].

### B. DBS Parameter Sampling Strategy

The following DBS parameter sampling strategy was used to acquire the fMRI maps in our dataset. For each patient, optimal voltage, frequency, pulse width and contact locations - a priori determined by the standard-of-care clinical protocol - were used for DBS-fMRI data collection. To generate non-optimal data points, sub-therapeutic and supra-therapeutic voltages, frequency (80 – 160 Hz) and contact locations that were tolerable to the patient were also used for DBS-fMRI data collection. The pulse width of the DBS parameters in our 39 patients cohort was kept constant at 60 ms. The number of data points per patient (maximum of 5 and minimum of 1) depended on how many supratherapeutic and subtherapeutic DBS parameter sets were tolerated by the patient. These tolerable DBS parameter combinations were not identical across all patients.

### C. Functional MRI Preprocessing

**FIGURE 2.**
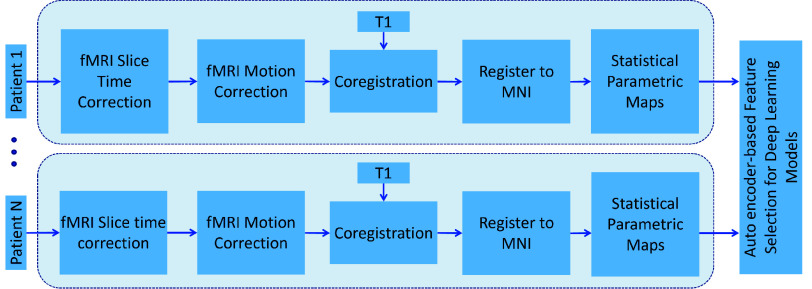
The acquired fMRI data from each patient are slice time corrected, motion corrected, coregistered to an anatomical (T1) image, registered to MNI space before carrying out a statistical parametric mapping analysis to obtain t-maps. The obtained t-maps are then passed to an auto-encoder network for feature learning.

All fMRI data analyses were carried out using SPM12 (https://www.fil.ion.ucl.ac.uk). The acquired fMRI data were slice time corrected, motion corrected, rigidly registered to a T1-weighted image, non-linearly registered to a standard space Montreal Neurological Institute (MNI) brain, and spatially smoothed using a Gaussian kernel with a 6 mm full width at half maximum (Figure 2). To account for artifacts due to patient head motion during data acquisition, we used the Art toolbox (https://www.nitrc.org/projects/ artifact detect) [19] to detect and remove volumes with motion >2 mm. Overall, for any given patient, this resulted in the removal of a maximum of 6 volumes (3.3%) from the total number of acquired volumes. Motion regression was implemented in the fMRI design matrix using 6-degrees of motion (x, y, z, yaw, pitch and roll) before statistical parametric maps were extracted from the data. The 6-degrees of motion parameters were correlated with DBS ON/OFF block design to ascertain that the observed changes were related to DBS stimulation paradigm, and not related to patient head-motion. Statistical parametric maps (t-maps) were estimated from the preprocessed fMRI data using the designed 30-second DBS-ON/OFF paradigm. The hemodynamic response function was modelled using the canonical double gamma function, as it was found to be similar to BOLD fMRI response of DBS across brain regions and patients [19], [20].

### D. Autoencoder-Based Feature Learning

To enhance the robustness of features extracted from DBS-fMRI response maps – for training and testing our DBS parameter classification and prediction models – we utilized an unsupervised AE-based feature extraction approach. The AE is a type of neural network model used for unsupervised learning and aimed at discovering underlying correlations within an input data and representing it in a lower-dimensional space. The AE employs a symmetrical network architecture primarily designed to encode the input data into a compressed and meaningful feature representation known as the latent space, and then decode it back such that the reconstructed input is similar or identical to the original input. A typical AE network is composed of two sub-networks popularly called encoder and decoder networks. The encoder is a compression function *E* that uses kernel weight, activation function, and bias ( $w, \delta \ and\ b$ respectively) to map the input data *x* to a lower dimensional latent space *z* [21], [22]. The decoder is a recovery function *D* that maps the latent space *z* to the output $x^{\prime } $ using kernel weight, activation function, and bias ( $w^{\prime }, \delta ^{\prime } \ and\ b^{\prime } $ respectively):

\begin{align*} z \gets E(x) & = \delta \left ({{wx + b}}\right) \tag{1} \\ x^{\prime } \gets D(z) & = \delta ^{\prime }\left ({{w^{\prime } h + b^{\prime }}}\right) \tag{2}\end{align*}

The goal of AE network is to learn the mapping functions *E* and *D*:

\begin{align*} & E: x \rightarrow z\ \text {(encoder)} \tag{3}\\ & D: z \rightarrow x^{\prime }\ \text {(decoder)} \tag{4}\end{align*}

that satisfy:

\begin{equation*} \arg \ {\min }_{E, D} \ {\mathbb {E}} \big [\mathcal {L}\big (x,\ D(E(x))\big)\big ] \tag{5} \end{equation*}

where $\mathbb {E}$ is the expectation over the distribution of input *x*, and $\mathcal {L}$ is the reconstruction loss function, which measures the difference between the original input and reconstructed input from the decoder [23]. The goal is to minimize the difference between the input and output reconstruction through the defined loss function.

During the AE training process, the loss function is optimized using gradient backpropagation. For simplicity and computational speed, $L_{2}$ loss, such as mean square error (MSE), is often chosen to compute the pixel-wise difference (Equation 6).

\begin{equation*} {\mathcal {L}}^{\mathrm {MSE}}\left ({{x,x^{\prime }}}\right) = \frac {1}{n}\sum _{i=1}^{n}{\Big (x_{i} -x^{\prime }_{i}\Big)^{2}}\tag{6} \end{equation*}

However, it is widely accepted that $L_{2}$ loss doesn’t correctly represent the human perception of image quality: $L_{2}$ simply does not capture the intricate characteristics of the human visual system [24]. In this work, a perceptual loss function based on the structural similarity index measure (SSIM) was used for the AE reconstruction loss. SSIM captures the difference in luminance, contrast, and structural information instead of simply computing pixel-wise differences. The SSIM is defined as [25]:

\begin{equation*} {\mathrm {SSIM}}(x,x^{\prime }) = {\frac {(2\mu _{x^{\prime }}\mu _{x}+c_{1})(2\sigma _{x^{\prime }x}+c_{2})}{(\mu _{x^{\prime }}^{2}+\mu _{x}^{2}+c_{1})(\sigma _{x^{\prime }}^{2}+\sigma _{x}^{2}+c_{2})}}, \tag{7}\end{equation*}

where $\mu _{x}^{\prime } $, $\sigma _{x}^{\prime } $ represent the mean and covariance for $x^{\prime } $ (similarly for *x*), $\sigma _{x^{\prime }x}$ is the covariance of $x^{\prime } $ and *x*. The values for $c_{1}$, $c_{2}$ stabilize the division with weak denominator. The SSIM-based loss function can then be defined as:

\begin{equation*} {\mathcal {L}}^{\mathrm {SSIM}}\left ({{x,x^{\prime }}}\right) = 1 - {\mathrm {SSIM}}(x,x^{\prime }) \tag{8}\end{equation*}

By optimizing this loss function, the network was able to reconstruct visually meaningful images that are perceptually similar to the original input images.

**FIGURE 3.**
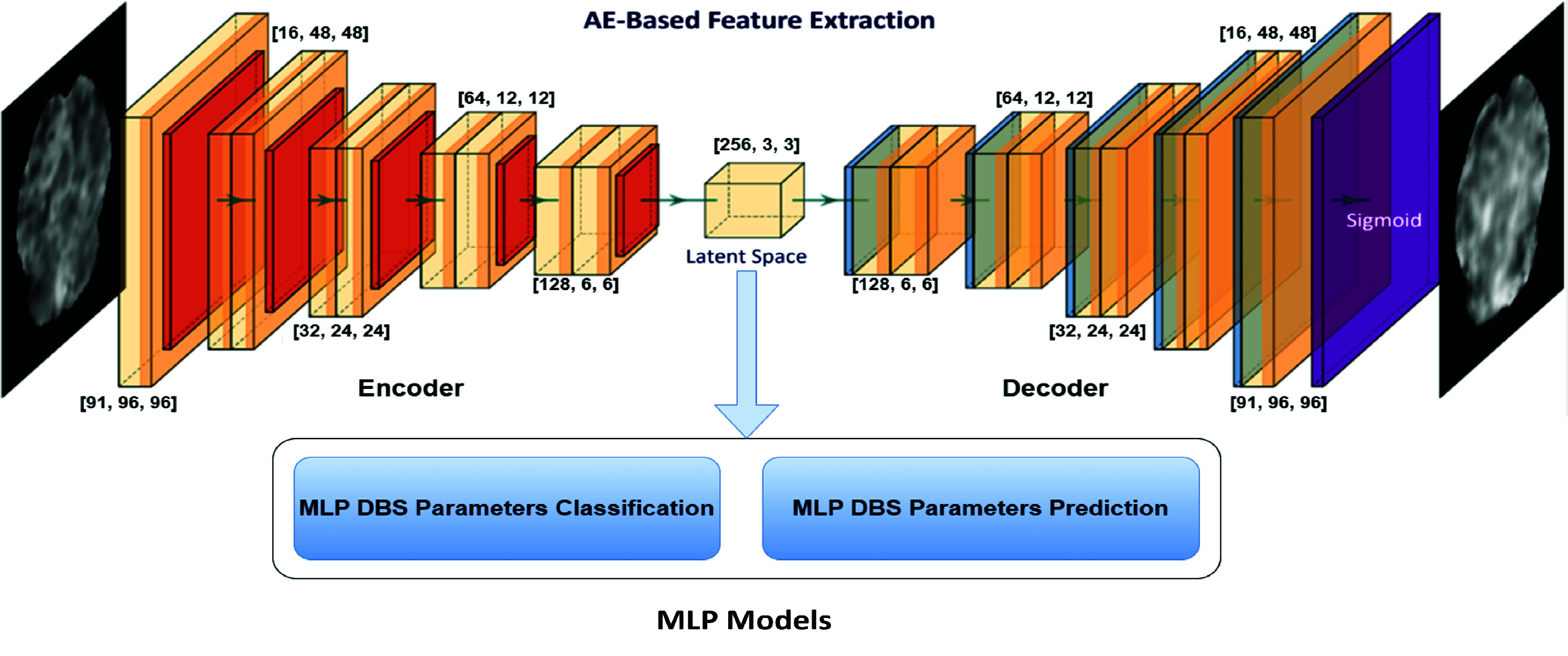
The network architecture for AE-based feature extraction from DBS-fMRI response maps (top). Numbers at each convolution block indicates the width, height, and depth of hidden feature map. The extracted features are then used to train MLP models (bottom) for DBS parameters classification and prediction.

To extract associative features from the fMRI response maps without prior information, we employed an unsupervised convolutional AE network, as shown in Figure 3. In our AE network implementation, the encoder part is composed of 6 convolution blocks, with each block consisting of a convolution layer, Rectified Linear Unit (ReLU) activation, and batch normalization. These convolution blocks employ filters to capture spatial patterns and extract relevant features from the input DBS-fMRI response maps. The numbers of filters in each convolution block are 1, 16, 32, 64, 128, and 256, respectively. The first convolution block utilizes a kernel size of 3 and a stride of 1. For downsampling, the subsequent convolution blocks use a kernel size of 4 and a stride of 2. The decoder part is responsible for upsampling and consists of 5 blocks, where each block is composed of a transposed convolution layer, ReLU activation, and batch normalization. The number of filters in each transposed convolution block are 128, 64, 32, 16, and 91, respectively. All transposed convolution layers use a kernel size of 4 and a stride size of 2.

This AE network facilitated the reduction of fMRI response maps from their initial dimensions of $91\times 96\times 96$ to compact $256\times 3\times 3$ latent space. To ensure compatibility with our AE network, all DBS-fMRI response maps were resized from the standard MNI dimensions of $91\times 91\times 109$ to $91\times 96\times 96$. Additionally, the resized fMRI response maps underwent normalization to a range between 0 and 1.

### E. Multilayer Perceptron for DBS Parameters Classification and Prediction

In brief, the MLP is a fully connected neural network renowned for its capacity as a universal approximator, capable of approximating virtually any smooth, measurable function [26], [27], [28]. Additional details regarding the MLP have been explained in previous studies [29], [30]. Our implementation of the MLP model comprised 8 hidden layers, with each neuron within a given hidden layer fully connected to all neurons in the subsequent layer. Each hidden layer (excluding the 8th layer) consisted of a linear layer followed by a ReLU activation function. To mitigate the risk of overfitting, four dropout layers were appended to the end of the first four blocks, with feature dropout percentages set at 25%, 15%, 15%, and 15%, respectively. The 8th layer functioned as a fully connected layer, mapping 16 neurons to 1 neuron, representing the probability of optimal DBS parameters in classification task, and the predicted optimal DBS parameter value in prediction task. For the classification model, a sigmoid activation function was employed at the last layer to obtain normalized probabilities.

### F. Model Training and Testing

Model training and testing were executed using Python 3.8 (Python Software Foundation, https://www.python.org/) and PyTorch 1.13.0 (https://www.pytorch.org/). The AE-MLP model training steps are described in Algorithm 1, which involves a two-stage training process. In the first stage, the AE network was trained for 250 epochs on the DBS-fMRI dataset using the SSIM-based loss function and the Adam optimizer with a learning rate of $1\times 10^{-4}$. In the second stage, the trained AE model from the first stage was utilized to extract latent features exclusively from the nominal DBS-fMRI response maps. These extracted features were subsequently employed to train the MLP classification and prediction models, aimed at determining the optimal DBS parameter settings. The MLP models underwent training for 100 epochs using binary cross-entropy (BCE) loss for classification tasks and MSE loss for prediction tasks. The Adam optimizer with a learning rate of $1\times 10^{-3}$ was used. During this stage, the trained AE model was in evaluation mode with fixed weights. Evaluation of the MLP classification and prediction models was conducted within a 5-fold cross-validation framework, where 80% of the entire dataset was allocated for model training, and the remaining 20% was reserved for testing, ensuring stratified partitioning.

Algorithm 1 **Training AE-MLP Model**
Require: DBS-fMRI training dataset: $(X, Y)$
Require: Epoch number for AE training: $\varphi $
Require: Epoch number for MLP training: $\beta $
Require: Number of folds for cross-validation: *k*
1: Train AE network *G* on *X* for $\varphi $ epochs with ${\mathcal {L}}^{\mathrm {SSIM}}$
2: Extract latent features from trained AE: $Z \gets ~G(X)$
3: Create *k*-fold cross-validation set: $(Z_{k}^{t}, Y_{k}^{t})$, $(Z_{k}^{v}, Y_{k}^{v})$
4: **for** $i= 1$, $i{+}{+}$, while $i < = k$ **do**
5: **for** $j= 1$, $j{+}{+}$, while $j < = \beta $ **do**
6: Train MLP classifier $C_{i}$ on $(Z_{i}^{t}, Y_{i}^{t})$ with ${\mathcal {L}}^{\mathrm {BCE}}$
7: Validate MLP classifier $C_{i}$ on $(Z_{i}^{v}, Y_{i}^{v})$
8: Save model weight with hightest accuracy.
9: **end for**
10: **for** $j= 1$, $j{+}{+}$, while $j < = \beta $ **do**
11: Train MLP predictor $P_{i}$ on $(Z_{i}^{t}, Y_{i}^{t})$ with ${\mathcal {L}}^{\mathrm {MSE}}$
12: Validate MLP predictor $P_{i}$ on $(Z_{i}^{v}, Y_{i}^{v})$
13: Save model weight with hightest accuracy.
14: **end for**
15: **end for**

The resulting AE-MLP classification model was used to classify DBS parameters as either optimal or non-optimal. The AE-MLP prediction model was used to predict the optimal contact location (x, y and z coordinates in MNI space), voltage and frequency. The MLP prediction model inherently learned the MNI coordinates of the Medtronic 3387 electrode contacts used in our cohort.

### G. Robustness of Autoencoder-Based Feature Extraction

We investigated the ability of the AE feature extraction model (trained on left-sided DBS data only) to extract disentangled features from DBS-fMRI response maps that contain changes in the activation patterns of the input response maps. To simulate variations in the DBS-fMRI responses due to differences in stimulation side and disease conditions, we applied a left-right flip operation to the original left-sided (nominal) response maps. This operation displaced the activated and deactivated regions horizontally, resulting in a new set of 122 flipped response maps. Subsequently, we extracted latent features from the flipped response maps using the same AE model trained on the nominal DBS-fMRI data for the sole purpose of comparison with the feature vectors extracted from the nominal DBS-fMRI maps. Furthermore, to understand the effects of the included GPi patient data on the trained models, we trained a separate classification and prediction model based on nominal DBS-fMRI responses of STN patients only (35 patients) using the same methods adopted for training and testing the STN-GPi models.

### H. Data Analyses and Visualization

The distribution of latent features extracted from both the nominal and flipped DBS-fMRI response maps were compared using violin plots and cosine similarity index (CSI). To visualize the latent features learned by the proposed AE feature extractor from the 122 DBS-fMRI datasets, we applied the t-Distributed Stochastic Neighbor Embedding (t-SNE) method [31], [32], which facilitates the visualization of high-dimensional data by mapping each data point to a location in a two-dimensional map. Metrics such as accuracy, precision, recall, F1 score, receiver operating characteristics (ROC) curve, precision-recall curve, and area under the curve (AUC) were use to assess the performance of the AE-MLP classification model. The performance of the AE-MLP prediction model was quantified using root mean square error (RMSE) and prediction accuracy at 10% and 15% tolerance levels (or deviation from the ground truth). Predictions were deemed accurate if they fell within ±10% and ±15%, respectively, from the ground truth. The performance of the AE-MLP prediction model was also visualized using a linear regression plot depicting the mean prediction values and their corresponding confidence limits.

## III. Results

**FIGURE 4.**
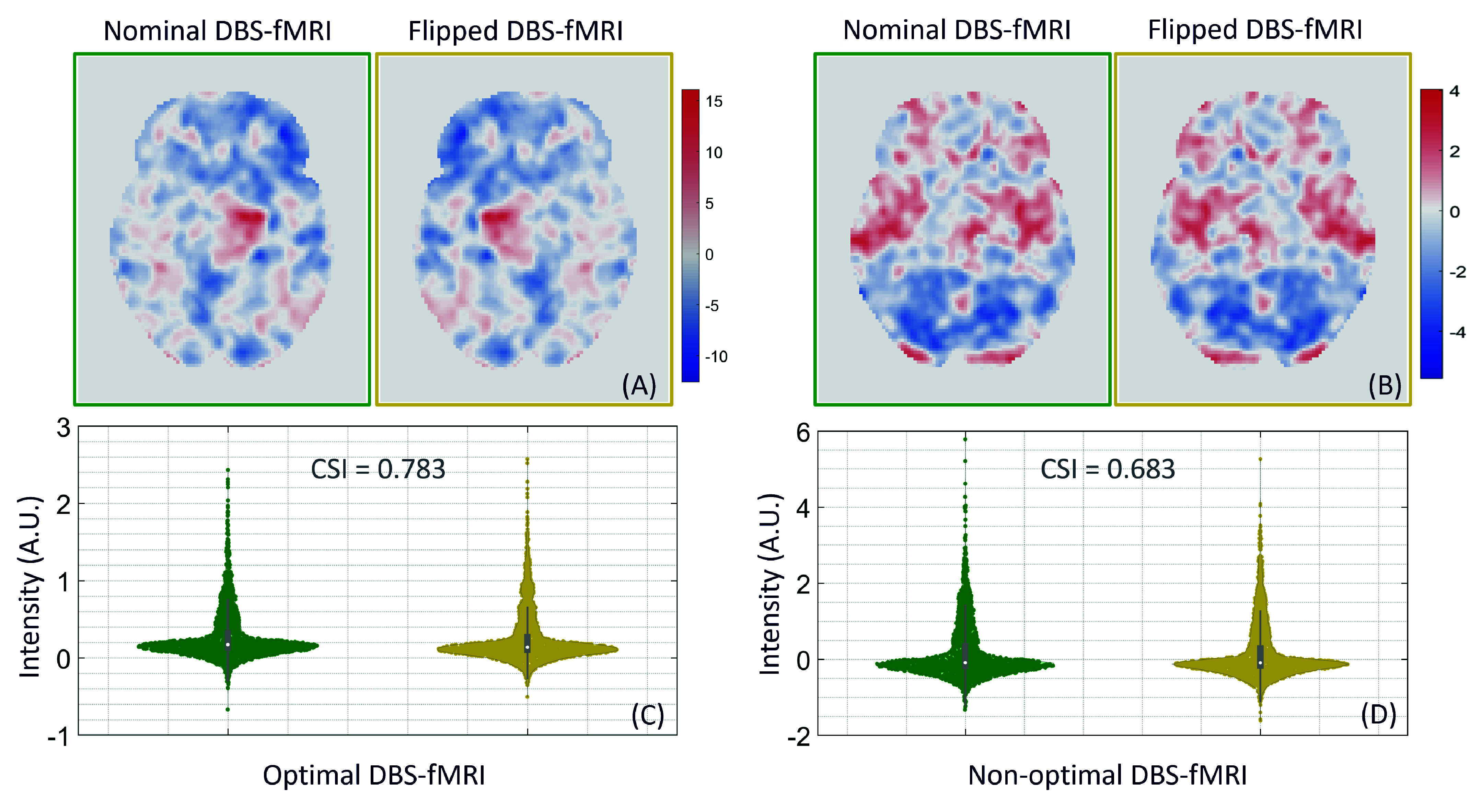
Axial view of representative nominal (green) and flipped (yellow) DBS-fMRI response maps at optimal (A) and non-optimal (B) stimulation parameters. The violin plots show the distribution of the features extracted by the AE model (trained on left or nominal DBS-fMRI responses) from the nominal and flipped responses at optimal (C) and non-optimal (D) stimulation parameters. The cosine similarity index (CSI) of the features extracted from the nominal and flipped response maps are also shown between the violin plots.

**FIGURE 5.**
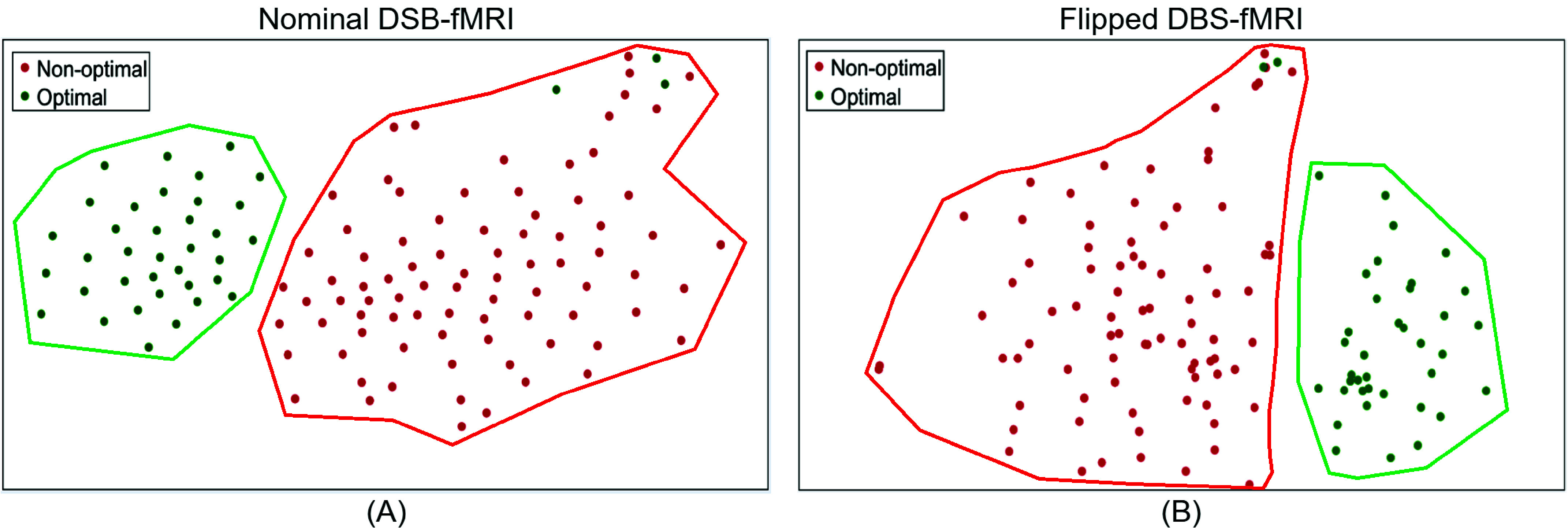
T-SNE visualization of the latent features extracted from the DBS-fMRI response maps (of the entire patient cohort) using the AE feature extraction model (trained on nominal or left DBS-fMRI responses only) indicates that the features obtained from the nominal (A) and flipped (B) DBS-fMRI response maps form clusters of optimal and non-optimal responses.

Representative nominal and flipped DBS-fMRI response maps, along with the distribution of AE-extracted features from optimal and non-optimal responses, are illustrated in Figure 4. Despite the differences in the topographic patterns of the nominal and flipped response maps, the distributions of their respective AE-extracted features were similar, with CSI values as high as 0.783 and 0.683 for the optimal and non-optimal response maps, respectively. The t-SNE visualization of the AE-extracted features for the entire patient cohort (122 data points) demonstrated significant clustering, effectively disentangling the optimal from non-optimal data points across both nominal and flipped DBS-fMRI response maps (Figure 5).

**FIGURE 6.**
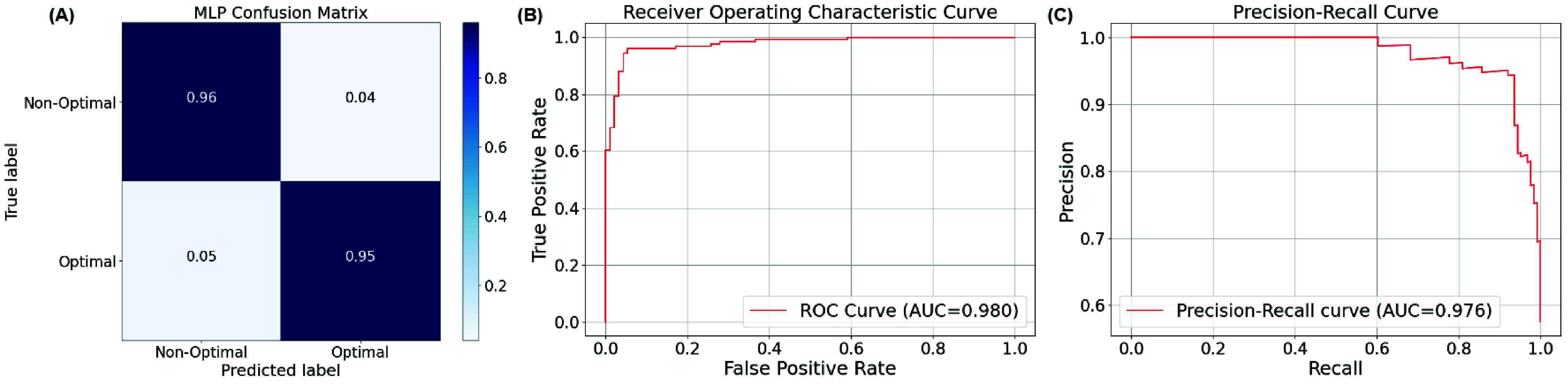
Combined confusion matrix (A), receiver operating characteristic (ROC) curve (B), and precision-recall curve (C) from 5-fold cross-validation for AE-MLP-based DBS parameters classification model.

The accuracy, precision, recall, and F1 score of the AE-MLP classification model trained on the STN-GPi dataset were 0.96 ± 0.04, 0.92 ± 0.07, 0.95 ± 0.07, and 0.93 ± 0.06, respectively. Compared to our previous PB-LDA method, which had an accuracy of 81%, the AE-MLP classification model demonstrates significantly improved performance. Figure 6 shows the combined confusion matrix, ROC curve, and precision-recall curves from the 5-fold cross-validation results for the AE-MLP classification model. The combined AUC of the ROC and precision-recall curves for the AE-MLP classification models were 0.980 and 0.976 respectively.

**FIGURE 7.**
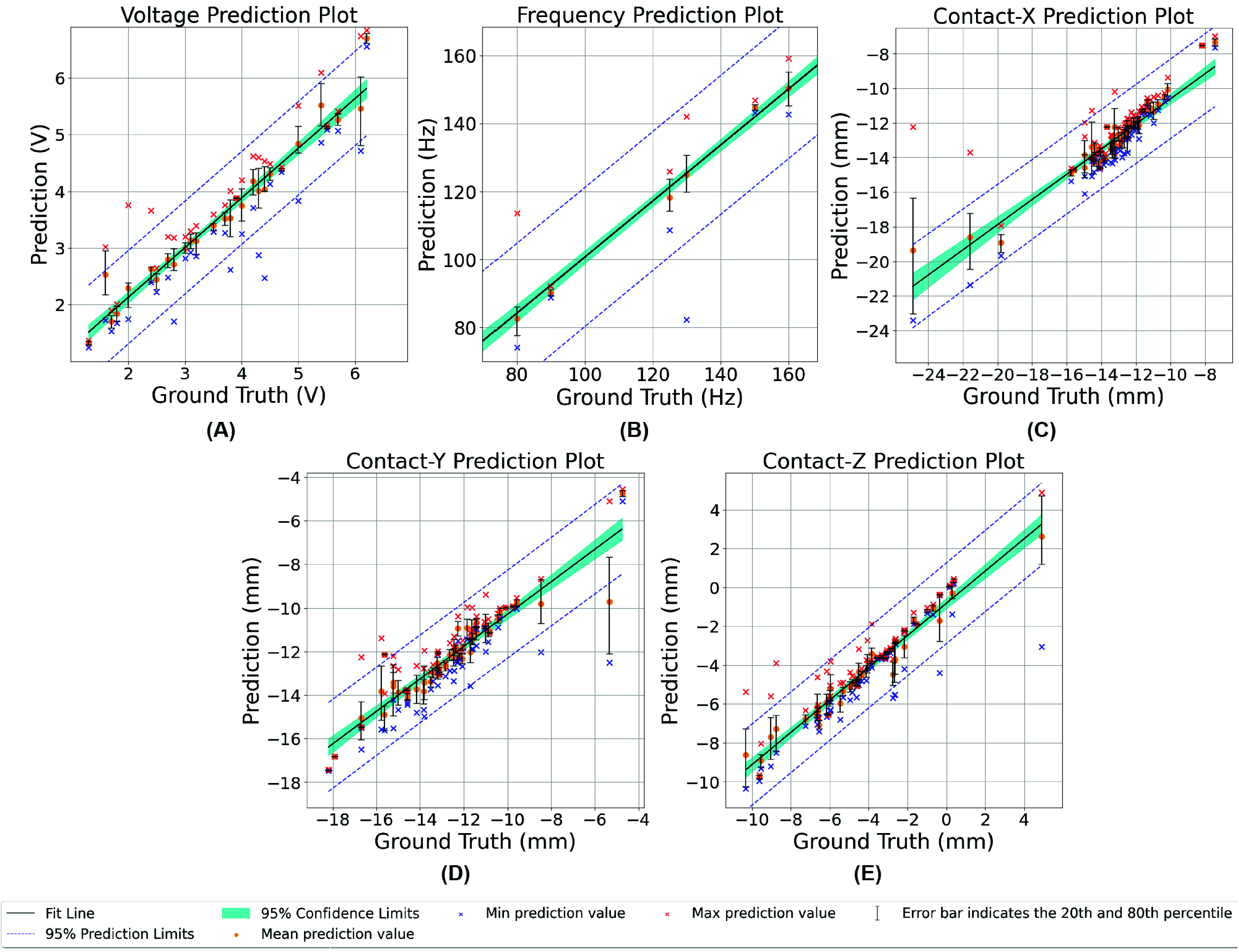
Comparison of the predicted and target optimal stimulation parameters including voltage (A), frequency (B), contact-x location (C), contact-y location (D), and contact-z location (E) for the AE-MLP-based DBS parameters prediction model.

The AE-MLP prediction model trained on the STN-GPi dataset achieved RMSE (from combined 5-fold cross-validation results) of 0.437 V, 12.493 Hz, 1.405 mm, 1.241 mm, and 1.163 mm for voltage, frequency, and x-y-z contact locations, respectively. A comparison of the predicted DBS parameters and target values are shown in Figure 7 for the voltage, frequency, and x-y-z contact locations. The accuracies of the AE-MLP prediction model at 10% and 15% tolerance (deviation from ground truth) are summarized in Table 1. As expected, the optimal DBS prediction accuracy at 15% tolerance was higher than the accuracy at 10% tolerance, with the lowest accuracy recorded in the prediction of z-location of the optimal contact.

**TABLE 1:** The Accuracy of the AE-MLP Model in the Prediction of DBS Voltage, Frequency, and x-y-z Contact Locations are Presented at 10% and 15% Deviation From the Ground Truth

DBS Parameter	Accuracy (10% Tolerance)	Accuracy (15% Tolerance)
Voltage (V)	0.79±0.04	0.91±0.03
Frequency (Hz)	0.85±0.04	0.89±0.04
Contract-X (mm)	0.82±0.05	0.94±0.04
Contract-Y (mm)	0.83±0.05	0.90±0.06
Contract-Z (mm)	0.70±0.07	0.76±0.08

The performance of the AE-MLP classification and prediction models trained on the STN-only dataset was comparable to that of the STN-GPi models. This similarity in performance may be associated with the limited number of GPi patients in our cohort. The accuracy, precision, recall and F1 score of the STN-only classification model was 0.97 ± 0.02, 0.95 ± 0.04, 0.95 ± 0.07 and 0.94 ± 0.04 respectively. The combined AUC of the ROC and precision-recall curves for this classification model were 0.982 and 0.980 respectively (see Supplementary Figure S1). The STN-only prediction model achieved a combined RMSE of 0.517 V, 13.93 Hz, and 1.27 mm, 1.16 mm, and 1.21 mm for voltage, frequency, and x-y-z contact locations, respectively. A comparison of the predicted DBS parameters and target values are shown in Supplementary Figure S2. The accuracies of this prediction model at 10% and 15% tolerance are summarized in Supplementary Table S1.

## IV. Discussion

In alignment with the recently renewed pursuit of biomarker-based semi-automated DBS optimization in PD patients, the unsupervised AE model has been utilized as a feature extraction method from DBS-fMRI response maps for MLP-based DBS parameters classification and prediction models. In theory, the entire DBS-fMRI response map, containing numerous features, can be used for building the classification and prediction model. However, doing so can rapidly render calculations laborious, less accurate, and time-consuming without a comprehensive understanding of the results [33].

The promising results of our AE-MLP classification and prediction models are desired metrics in the drive for rapid semi-automated DBS optimization. The utilization of AE-extracted features in the current MLP-based DBS parameters classification yielded a significant increase in overall accuracy from 81% (as achieved in our previous PB-LDA implementation [11]) to 96%, thereby highlighting the important role of an effective and robust feature extraction method. The RMSE of the AE-MLP prediction model yielded values that are within tolerable clinical limits. For example, the RMSE in the prediction of the voltage was 0.437 V, which is within the commonly-used voltage adjustment step-size in the standard-of-care clinical optimization protocol [3], [4].

The efficacy of a single AE model (trained using only left-sided or nominal DBS-fMRI responses) is evident in the disentanglement of extracted optimal and non-optimal features, as depicted in the t-SNE plots for the nominal and flipped response maps. The t-SNE visualizations demonstrated disentanglement because the DBS-fMRI map and their corresponding parameter sets – assessed as clinically optimal were clustered differently from those determined to be clinically non-optimal. These disentangled features enhance the classification and prediction of the optimal DBS parameter settings from their corresponding response maps. The activation and deactivation pattern variability in the optimal and non-optimal DBS-fMRI data are well captured in the latent features extracted by the AE feature-extraction model.

Although our left-right flipping of the nominal DBS-fMRI response maps to generate the flipped responses does not precisely capture the real-world differences that will be imposed on a DBS-fMRI acquisition by differences in stimulation side or disease condition, these results indicate that the AE-based feature extraction method is potentially robust to differences in response maps that change the activated and deactivated regions. Our results also suggest that the left-right flipping operation may be used for data augmentation of single-sided DBS-fMRI data, which is potentially useful for training more robust DL models for DBS parameter optimization.

Though bold response to chronic DBS is likely not the same as bold response during acute DBS [34], our current 30-second DBS on/off cycling stimulation fMRI paradigm was aimed at capturing the hemodynamic response of the applied DBS stimuli, and to determine if the captured bold response can serve as a biomarker to differentiate between optimal and non-optimal DBS parameter sets using long-term clinical outcomes as ground truth for optimal parameters. This rapid approach is crucial as the desired utility of our research output is to have a rapid fMRI biomarker-based prediction of optimal DBS parameters that yield improved chronic clinical outcomes. Our group has previously shown the efficacy of the current fMRI paradigm to capture the bold biomarkers that discriminate between optimal and non-optimal DBS parameters in Parkinson’s disease, dystonia, and depression [11], [17], [35].

A limitation of this work is the imbalance in the available data, with more non-optimal than optimal DBS-fMRI responses. However, the use of the unsupervised AE-based feature learning method may have mitigated the impact of this data imbalance on the obtained results because the extracted features are well clustered in the t-SNE plot. Additionally, the high F1 score of the models in 5-fold cross-validation suggests that the data imbalance is well-tolerated by the AE-MLP classification methods. The smaller number of GPi patients in our dataset (4 GPi compared to 35 STN DBS patients) may also affect the generalizability of our models to this patients population, as the limited number of GPi patients may mean that the models are not able to adequately capture the variability and distinct fMRI responses associated with GPi DBS.

Another drawback of this work is the limited number of available datasets for model training and validation, attributable to the relatively small number of PD patients able to access DBS therapy [36], [37], [38], [39], [40], [41], [42]. We acknowledge that the small size and limited diversity of our dataset may reduce the accuracy of the current AE-MLP prediction model when deployed for optimizing newer DBS electrodes with additional directionality parameters. As we continue to gather more DBS-fMRI data from directional DBS electrodes, we anticipate an improvement in the performance of the prediction model. These preliminary results demonstrate the effectiveness of our DBS parameter prediction model, which has the potential to reduce the time to optimization from approximately a year to a few hours during a single clinical visit. This is particularly timely given the ongoing implementation of new directional DBS electrodes in clinical practice.

The present data were used to investigate fMRI brain changes associated with different DBS contact location, voltage and frequency settings. The availability of fMRI data with variable DBS pulse width parameter will facilitate a more robust prediction model for DBS parameter optimization. As previously demonstrated, fMRI is an effective biomarker of optimal DBS stimulation [11]. Such a biomarker-based programming tool could be leveraged to decrease the TPP and the number of clinic visits required before DBS patients’ optimal settings are identified. This is particularly important as the number of possible stimulation parameters increases with modern DBS electrodes, which have been reported to be more effective with a broader therapeutic window [43]. The expanded parameter space of modern DBS electrodes has made it impractical for clinicians to perform DBS parameter optimization manually within a clinically acceptable timeframe, thereby hindering the adoption of these newer and more effective electrodes. Furthermore, programming could theoretically be performed in the absence of specialized DBS physicians in non-expert centers.

**FIGURE 8.**
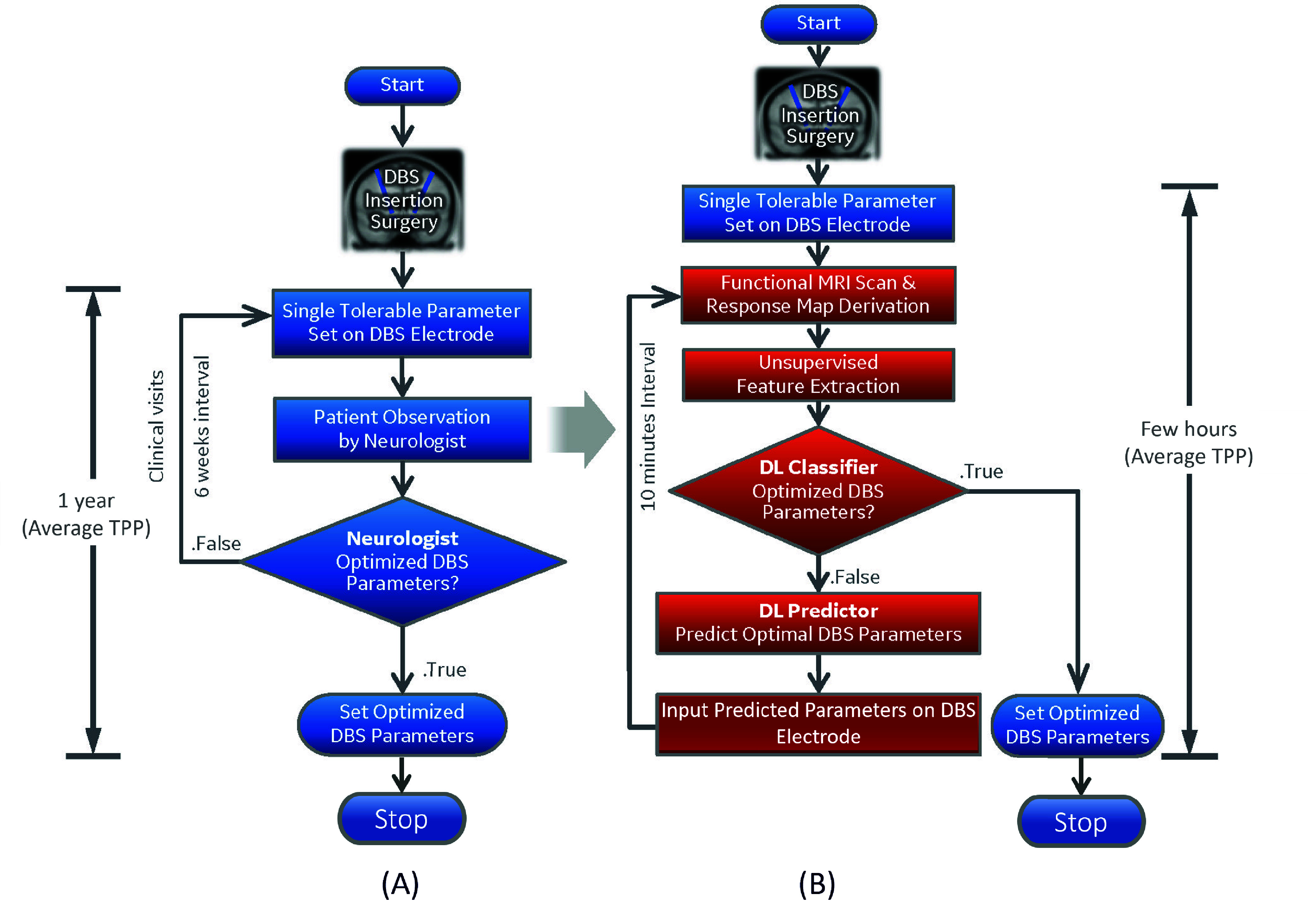
(A) In the current empirical programming of DBS electrodes after the insertion surgery, the programming parameters (contact, voltage, frequency, and pulse width) are manually and sequentially adjusted until an optimal parameter combination is reached as determined by the neurologist. (B) In the proposed semi-automated optimization protocol, the features extracted from the DBS-fMRI response map of a patient are passed through the DL-based classifier to determine if the set of DBS parameters used to obtain the bold responses is optimal. If optimal, the DBS parameters are set by the attending clinician and the optimization is ended. If the DL-based classifier determines that the DBS parameters are non-optimal, the extracted latent features are passed through the MLP-based prediction model to predict the optimal DBS parameters. The predicted optimal parameters are used to obtain another bold response, whose latent features are passed through the DL classifier. The loop is terminated if the parameters are classified as optimal and the clinician sets the optimal parameters. Such rapid semi-automated DBS programming protocol can substantially reduce the TPP from an average value of 1 year to a few hours during a single clinical visit.

Finally, the proposed AE-MLP-based DBS parameters classification and prediction pipeline represent another step towards translating a digital healthcare tool for semi-automated fMRI-based DBS programming. As such, we propose a rapid semi-automated DBS programming protocol that can substantially reduce the TPP from an average value of 1 year (based on the current standard-of-care clinical optimization procedure) to a few hours during a single clinical visit (see Figure 8). Such automatic DBS optimization systems could be transformative in diseases where there is a latency in clinical feedback (e.g., dystonia) [17], or where clinical responses are difficult to evaluate (e.g., depression) [35], [44]. Together, these benefits could dramatically increase the number of patients able to benefit from DBS therapy worldwide, while minimizing the time and financial burden needed for DBS optimization per patient.

## V. Conclusion

Since fMRI has previously been shown to be a good biomarker of optimized DBS parameter settings, we developed an AE-based model for unsupervised feature extraction from DBS-fMRI response maps obtained from PD patients undergoing left-sided DBS therapy. We showed that the AE-extracted features were disentangled across optimal and non-optimal stimulation with robustness against differences in the activated regions that may be caused by differences in disease condition and/or stimulation side during the acquisition of the training data. We then evaluated the performance of the extracted features in MLP-based DBS parameter classification and prediction models. The AE-MLP classification method represents a significant improvement over our previously developed PB-LDA classification method. Furthermore, the AE-MLP based prediction method showed promising results for fMRI-based DBS parameter optimization. We then proposed a semi-automated schema for DBS parameter optimization that utilizes the AE-MLP classification and prediction models to potentially reduce the TPP from an average of 1 year to a few hours during a single clinical visit.

## Supplementary Materials

Supplementary materials
