# Supplementary material for: Deep Learning and fMRI-Based Pipeline for Optimization of Deep Brain Stimulation During Parkinson’s Disease Treatment: Toward Rapid Semi-Automated Stimulation Optimization
Source: IEEE J Transl Eng Health Med. 2024 Aug 22;12:589–99. doi: 10.1109/JTEHM.2024.3448392 (PMC11379443; doi:10.1109/JTEHM.2024.3448392)
Supplement: Supplementary materials [file supp1-3448392.pdf]

Deep Learning and fMRI-based Pipeline for Optimization of Deep Brain Stimulation During Parkinson's Disease Treatment: Towards Rapid Semi-automated Stimulation Optimization

Jianwei Qiu[1,†], Afis Ajala[1,†,*], John Karigiannis[2], Jürgen Germann[3,4], Brendan Santyr[3], Aaron Loh[3], Luca Marinelli[1], Thomas Foo[1], Radhika Madhavan[1], Desmond Yeo[1], Alexandre Boutet[3,4], Andres Lozano[3,4,5,6]

***Keywords*—Deep brain stimulation optimization, deep learning, fMRI, Parkinson's disease, unsupervised feature extraction**

***Clinical and Translational Impact Statement*—**A deep learning-based pipeline for semi-automated DBS parameter optimization is presented, with the potential to significantly decrease the optimization duration per patient and patients' financial burden while increasing patient throughput.

[1]GE HealthCare Technology & Innovation Center, Niskayuna, NY 12309, United States of America
[2]GE Global Research, Niskayuna, NY 12309, United States of America
[3]Division of Neurosurgery, Department of Surgery, University Health Network and University of Toronto, Toronto, ON, Canada
[4]Joint Department of Medical Imaging, University of Toronto, Toronto, Canada
[5]Krembil Brain Institute, Toronto, ON, Canada
[6]Center for Advancing Neurotechnological Innovation to Application (CRANIA), Toronto, ON, Canada.
*Corresponding authors: A. Ajala (afis.ajala@gehealthcare.com)
†These authors contributed equally to this work

SUPPLEMENTARY MATERIALS

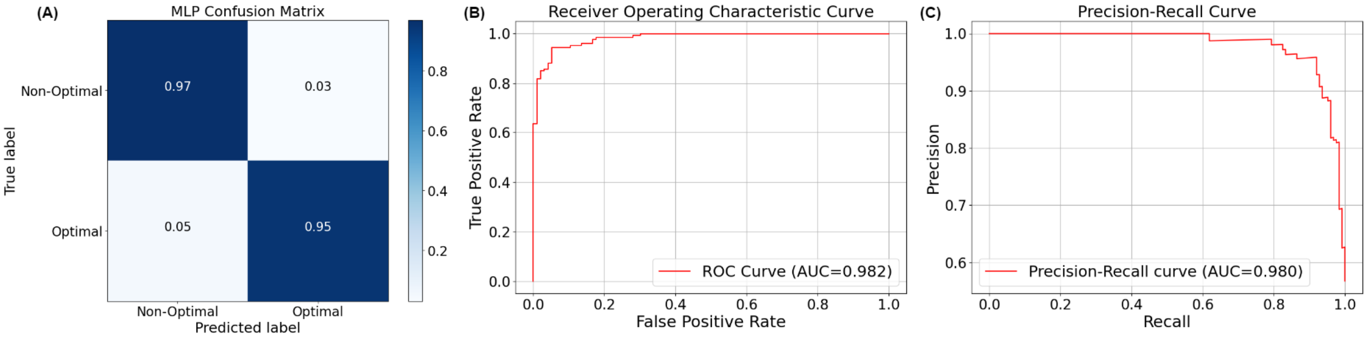

Fig. S1. Combined confusion matrix (A), receiver operating characteristic (ROC) curve (B), and precision-recall curve (C) from 5-fold cross-validation for AE-MLP-based DBS parameters classification model trained on the STN-only dataset.

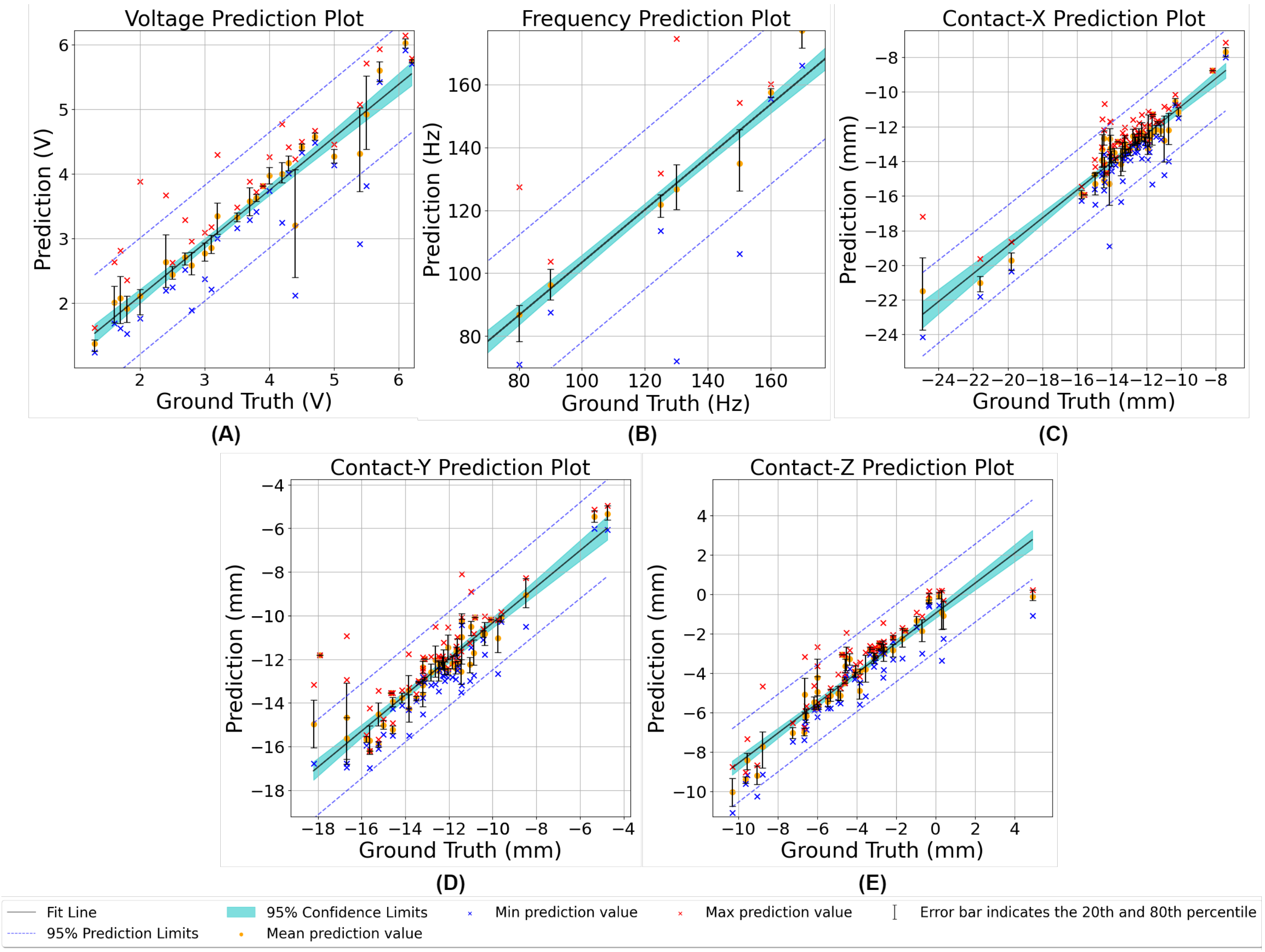

Fig. S2. Comparison of the predicted and target optimal stimulation parameters including voltage (A), frequency (B), contact-x location (C), contact-y location (D), and contact-z location (E) for the AE-MLP-based DBS parameters prediction model trained on the STN-only dataset.

TABLE S1
THE ACCURACY OF THE AE-MLP PREDICTION MODEL, TRAINED ON STN-ONLY DATASET, IN FORECASTING OPTIMAL DBS VOLTAGE, FREQUENCY, AND X-Y-Z CONTACT LOCATIONS ARE PRESENTED AT 10% AND 15% DEVIATION FROM THE GROUND TRUTH.

**DBS Parameter**	**Accuracy (10% Tolerance)**	**Accuracy (15% Tolerance)**
Voltage (V)	0.77±0.06	0.88±0.05
Frequency (Hz)	0.83±0.05	0.87±0.04
Contract-X (mm)	0.85±0.04	0.93±0.03
Contract-Y (mm)	0.84±0.06	0.91±0.04
Contract-Z (mm)	0.67±0.08	0.74±0.08
